# Ultrasound-guided bilateral greater occipital nerve block for the treatment of post-dural puncture headache

**DOI:** 10.12669/pjms.311.5759

**Published:** 2015

**Authors:** Fethi Akyol, Orhan Binici, Ufuk Kuyrukluyildiz, Guldane Karabakan

**Affiliations:** 1 Fethi Akyol, M.D. Erzincan University Faculty of Medicine, Gazi Mengucek Education and Research Hospital, Anesthesiology and Reanimation, Erzincan, Turkey.; 2 Orhan Binici, M.D. Erzincan University Faculty of Medicine, Gazi Mengucek Education and Research Hospital, Anesthesiology and Reanimation, Erzincan, Turkey.; 3 Ufuk Kuyrukluyildiz, M.D. Erzincan University Faculty of Medicine, Gazi Mengucek Education and Research Hospital, Anesthesiology and Reanimation, Erzincan, Turkey.; 4 Guldane Karabakan, M.D. Erzincan University Faculty of Medicine, Gazi Mengucek Education and Research Hospital, Anesthesiology and Reanimation, Erzincan, Turkey.

**Keywords:** Greater occipital nerve, Post-dural pain headache, Ultrasound

## Abstract

**Background and Objective:**

Post-dural puncture headache (PDPH) is one of the complications frequently observed after spinal or epidural anesthesia with dural penetration. For PDPH patients who do not respond to conservative medical treatment, alternative treatments such as bilateral occipital nerve block should be considered.In this study the efficacy of bilateral occipital nerve block was retrospectively evaluated in patients with post-dural puncture headache.

**Methods:**

Ultrasound-guided bilateral occipital nerve block was administrated in 21 patients who developed PDPH after spinal anesthesia, but did not respond to conservative medical treatment within 48 hours between January 2012 and February 2014. The study was conducted at Erzincan University Faculty of Medicine Gazi Mengucek Education and Research Hospital

**Results:**

Mean Visual Analog Scale (VAS) pain scores at 10 minutes and 6, 10, 15 and 24 hours after the block were significantly improved compared to the patients with a pre-block VAS score between 4 and 6 as well as patients with a pre-block VAS score between 7 and 9 (p<0.01). After 24 hours of the block applied, VAS pain score dropped to 1 for all 12 patients who had a pre-block VAS score between 4 and 6. Whereas, VAS score decreased to 2 at 24 hours after the block in only one of the patients with a pre-block VAS between 7 and 9. For the patients with a pre-block VAS score between 7 and 9, there was no significant improvement in the mean VAS score 24 hours after the block.

**Conclusions:**

For patients with PDPH and a pre-block VAS score between 4 and 6 who do not respond to conservative medical treatment, an ultrasound-guided bilateral occipital nerve block may be effective.

## INTRODUCTION

Post-dural puncture (PDPH) headache is a common complication for patients with neuroaxial anesthesia.^1^ The International Headache Society defines PDPH as pain that may be bilateral and starts within 7 days and ends within 14 days, developing following a lumbar puncture.^2^ PDPH develops due to a loss of cerebrospinal fluid (CSF) from the location of the dural rupture towards the epidural area. The sudden decrease in CSF causes the development of an inflammatory reaction in sensitive structures such as the dura mater, cerebral arteries and venous sinus, leading to PDPH.^3^ The classical symptoms of PDPH are photophobia, nausea, vomiting, neck stiffness, tinnitus, double vision, dizziness and severe, throbbing headache. The headache begins at the occipital lobe and spreads to the frontal regions, eventually reaching the neck and shoulders, and intensifies with standing.^4^^,^^5^ The greater occipital nerve penetrates the semispinal iscapitis & trapezius muscles to innervate the skin along the posterior portion of the scalp to the vertex of the skull and the scalp over the ear and parotid glands.^6^^,^^7^ It takes sensorial tendons from the C2 and C3 segments of the spinalis. It separates from the dorsal ramus of the C2 segment, takes a fine branch from the C3 segment and innerves the posterior medial of the scalp to the anterior of the vertex. A greater occipital nerve block prevents the sense of pain in this region.^8^

In this study we evaluated the PDPH cases that underwent bilateral greater occipital nerve block, who were referred to Erzincan University Faculty of Medicine Gazi Mengucek Education and Research Hospital, and their response to the therapy.

## METHODS

This retrospective study assessed the effect of a bilateral greater occipital nerve block administered in 21 patients, all American Society of Anesthesiology Risk Classification I or II, who developed PDPH after receiving spinal anesthesia between February 2012 and January 2014 at the Erzincan University Faculty of Medicine Gazi Mengucek Education and Research Hospital. The study was approved by the Erzincan University Faculty of Medicine Ethical Assessment Commission for the Researches on Human (letter dated 18.02.2014 and numbered 01/11), and the required ethical committee permit was obtained. The patients ranged in age from 19 to 63. The patients with hemorrhagic diathesis, a history of past head trauma, neurological headache anamnesis or cranial defects were excluded from the study. Patient information was obtained by reviewing the patient files and anesthesia observation forms, and the pain scores were obtained by talking with the patients in person after the intervention.

**Fig.1:**
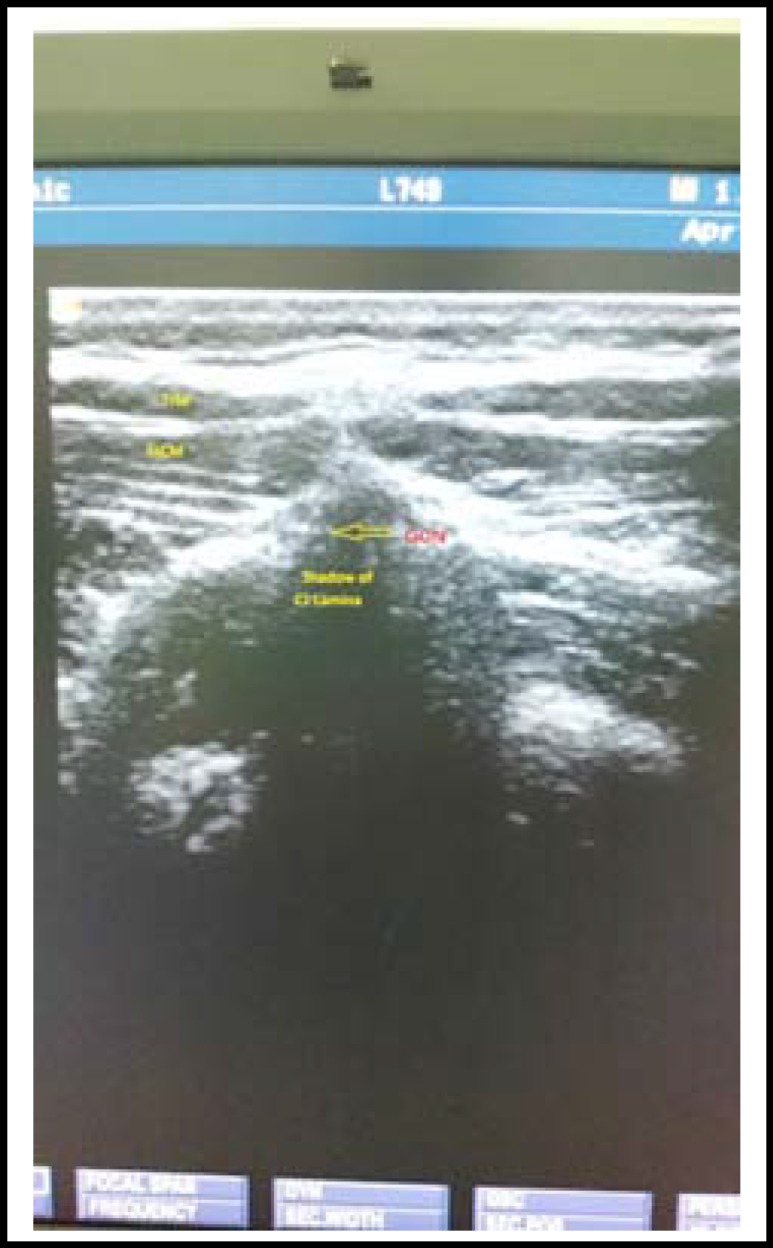
TrM, Trapezius Muscle; SsCM, Semispinalis Capitis Muscle; GON, Greater Occipital

**Fig.2:**
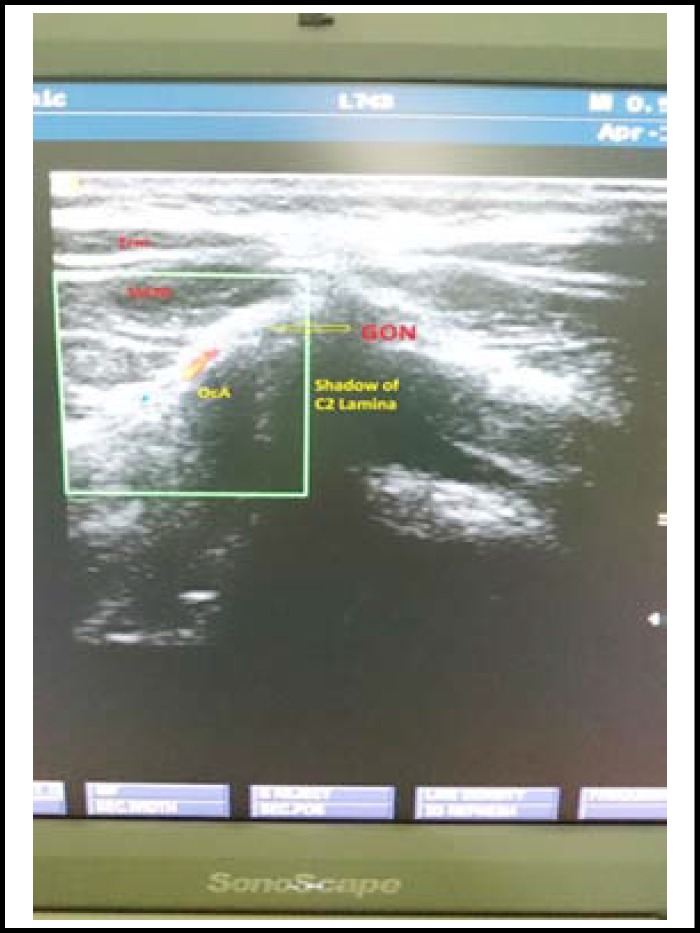
TrM, Trapezius Muscle; SsCM, Semispinalis Capitis Muscle; GON, Greater Occipital Nerve; OcA, Occipital Arter

Following administration of spinal anesthesia, up to 48 hours of bed rest together with oral or intravenous fluid and analgesics with caffeine were recommended for the patients with PDPH. For the patients with a Visual Analog Scale (VAS) pain score of 4 or above, an ultrasound guided bilateral greater occipital nerve block was administered with 4 mL 0.25% levobupivacaine injected lateral to the nuchal’s medial line, directly medial to the occipital artery. (Fig.1 & Fig.2) Age, sex, surgery indication, ASA values, complications developed during and after the intervention and VAS pain scores at 10 minutes and 6, 10, 15, and 24 hours after intervention were obtained in all the patients from the recordings. By the end of the first 24 hours after occipital block, the subjects with a VAS score of 3 or above were treated with alternative invasive methods.

***Statistical analyses: ***The statistical analyses were conducted using SPSS v17.0 software. Descriptive statistics such as the frequency, arithmetic mean, standard deviation, median and percentage were calculated. For the statistical comparisons, the non-parametric Mann-Whitney U, Wilcoxon Signed Rank and Chi Square tests were used. A p value < 0.05 was considered statistically significant.

## RESULTS

Before the block was applied, 12 of the patients had a VAS score between 4 and 6, and nine of the patients had a VAS score between 7 and 9. Of the 21 subjects, 61.9% (n=13) were male and 38.1% (n=8) were female. An equal number of males and females had a VAS score between 4 and 6 before the block was applied. Of the patients with an initial VAS score between 7 and 9, 77.8% (n=7) were male and 22.2% (n=2) were female. There are no statistically significant differences between the sexes of those with VAS value between 4-6 and between 7-9 before block. (p=0.367).

The age range of the subjects included in the study was 19-63 (mean= 36.95±14.42, median=32). The age range of those with a VAS score between 4 and 6 before the block was 19-63 (mean=35.58±16.67, median=29).The age range of those with a VAS value between 7 and 9 before the block was 25-62 (mean=38.78±11.45, median=36). There were no significant difference between the ages of those patients with an initial VAS score between 4 and 6 and those with an initial VAS score between 7 and 9 (p=0.270).

***Distribution by ASA Value: ***Of the total patients, 81% (n=17) were classified as ASA1and 19% (n=4) were ASA2. Of those patients with a VAS score between 4 and 6 before the block, 75% (n=9) were ASA1 and 25% (n=3) were ASA2.Of those patients with a VAS score between 7 and 9 before the block, 88.9% (n=8) were ASA1 and 11.1% (n=1) were ASA2. There was no significant difference in the distribution of patients classified as ASA1 and ASA2 for patients with a VAS score between 4 and 6 compared with patients with a VAS between 7 and 9 before the block (p=0.603).VAS Scores at Different Time Points Post-Block.

Table-I shows the mean +/- standard deviation, median, minimum and maximum VAS scores before the block and at time points after the block was applied according to the pre-block VAS score range. The Wilcoxon Signed Rank test was used to determine if there were any significant differences in VAS scores before the block and at 10 minutes or 6, 10, 15 or 24 hours after the block. The results are shown in Table-II. For both the group of patients with a pre-block VAS score between 4 and 6 and those with a pre-block VAS score between 7 and 9, there was a significant difference between the initial VAS score and the VAS scores at all of the time points after the block (p<0.01). Twenty-four hours after the block, all 12 patients with a pre-block VAS score between 4 and 6 had a VAS score of 1, but only a single patient with a pre-block VAS score between 7 and 9 reached a VAS of 1 by 24 hours. For the patients with a pre-block VAS score between 7 and 9, the mean VAS score 24 hours after the block was 5.56; thus, most of these patients did not recover from PDPH within 24 hours.

Table-III shows the numbers of patients who recovered within 24 hours according to the VAS score group. Those with a VAS score of 1 were considered to have recovered. The percentage of patients with a pre-block VAS score between 4 and 6 who recovered within 24 hours was significantly different than the percentage of patients with a pre-block VAS score between 7 and 9 (p<0.01).

## DISCUSSION

The incidence of PDPH is higher in younger patients, women, subjects with multiple holes in the dura and when quincke needles are used.^9^ In the literature, the incidence of PDPH after the use of a 25 G quincke needle is reported to be 3-25% and the use of a 25 G whitacre is 0-14.5%.^10^^,^^11^ Since we used a 25 G quincke spinal needle for all of the patients in this study, we projected that the PDPH incidence would be increased in these patients accordingly. The following treatments are used to treat PDPH: oral orintravenous fluids and analgesics with caffeine, microcatheter application to the spinal gap, epidural blood patch and fiber optical imaging mediated epidural interventional techniques.^12^

The nociceptive stimulations arising from the meninx in the cervical region causes sensitization in convergent neurons in the back horn at the C2 level. Blocking the greater occipital nerve blocks the stimulation generated from the innervated regions, the deep paraspinal muscles of the branch arising from the C2 root of the greater occipital nerve and the suboccipital structures.^13^ The indication of greater occipital nerve blockage is not explicit or clear, but it is administered for cervical headache, cluster headache, occipital neuralgia and migraine.^14^ The greater occipital nerve is located in the medial of the occipital artery at the superior nuchal level. The blockage of this nerve is possible by determining the occipital artery with trans-cranial ultrasonic Doppler.^15^

Because the greater occipital nerve has a superficial settlement, its blockage has few complications; however, there is a risk of intravenous injection, which can be prevented by a cautious aspiration.^16^ In our practice, we aspirate before injecting the medication from the medial by displaying the occipital artery with ultrasound. We did not encounter any complications in the patients during or after this block.

A previous case report of bilateral occipital nerve blocking applied in a safe manner in two patients with PDPH found that the patients’ pain stopped within several minutes.^8^ Similarly, in our analysis, the VAS score dropped to 1 within 10 minutes for 7 of the patients. In another case report, bilateral occipital nerve blocking completely relieved a patient’s PDPH within 2 minutes, but the pain reappeared 12 hours later; the block was then repeated.^17^ In our study, the bilateral occipital nerve block was administered only once. The PDPH of all the subjects with a pre-block VAS score between 4-6 was gone 24 hours after the block. Of the patients with a pre-block VAS score between 7 and 9, only 1 subject was completely recovered at 24 hours after the block. The rest of these patients experienced a decrease in PDPH, but the pain then increased again; a bilateral occipital nerve block was not repeated on these patients.

In a randomized, controlled study involving 50 patients with PDPH, 68% of the patients experienced full analgesia with the first or second bilateral occipital block; the pain management of the patients were more successful compared with the control group and the hospitalization periods were shorter.^18^ In our study, 57 % of the patients experienced full analgesia with a single bilateral occipital nerve block.

**Table-I:** Descriptive statistics according to pre-block VAS score group

**VAS Group**	**n=12**	**n**	**Mean**	**S.D.**	**Median**	**Min.**	**Max.**
Between 4 - 6	VAS Before Block	12	4.75	.866	4.50	4	6
	VAS 10th minute	12	1.50	.522	1.50	1	2
	VAS 6th Hour	12	1.00	.000	1.00	1	1
	VAS 10th Hour	12	1.08	.289	1.00	1	2
	VAS 15th Hour	12	1.17	.389	1.00	1	2
	VAS 24th Hour	12	1.00	.000	1.00	1	1
VAS Group	n=9	n	Mean	S.D.	Median	Min.	Max.
Between 7 - 9	VAS Before Block	9	7.78	.833	8.00	7	9
	VAS 10th minute	9	2.22	.667	2.00	1	3
	VAS 6th Hour	9	1.22	.441	1.00	1	2
	VAS 10th Hour	9	2.78	1.394	3.00	1	5
	VAS 15th Hour	9	5.11	1.616	6.00	1	6
	VAS 24th Hour	9	5.56	1.810	6.00	1	7

**Table-II:** Comparison of VAS scores before and after the block according to the pre-block VAS score group

**VAS Group**	**n=12**		**Mean**		**Z**	**P**
Between 4 - 6	VAS Before Block	VAS 10th minute	4.75	1.50	-3.108	.001
	VAS Before Block	VAS 6th Hour	4.75	1.00	-3.111	.000
	VAS Before Block	VAS 10th Hour	4.75	1.08	-3.115	.001
	VAS Before Block	VAS 15th Hour	4.75	1.17	-3.097	.000
	VAS Before Block	VAS 24th Hour	4.75	1.00	-3.111	.000
	n=9		Mean		Z	P
Between 7 - 9	VAS Before Block	VAS 10th minute	7.78	2.22	-2.724	.004
	VAS Before Block	VAS 6th Hour	7.78	1.22	-2.724	.004
	VAS Before Block	VAS 10th Hour	7.78	2.78	-2.699	.003
	VAS Before Block	VAS 15th Hour	7.78	5.11	-2.699	.004
	VAS Before Block	VAS 24th Hour	7.78	5.56	-2.699	.005

p: Monte Carlo Sig. (2-tailed)

**Table-III:** The recovery status of the patients 24 hours after the block according to the pre-block VAS score group

**VAS group**		**VAS 24th Hour**		**Total**
		**Recovered**	**Not recovered**	
Between 4 - 6	n	12	0	12
	%	100.0%	0.0%	100.0%
Between 7 - 9	n	1	8	9
	%	11.1%	88.9%	100.0%
Total	n	13	8	21
	%	61.9%	38.1%	100.0%

Fisher's Exact Test; p=0.000

Although an epidural blood patch can be used as an effective treatment for PDPH, we prefer the ultrasound-guided bilateral occipital nerve blockage, because it is easier to perform and has fewer complications. The epidural blood patch is invasive and is associated with potential complications such as neurological sequel, radiculopathy, spinal-subdural hematoma, spinal-epiarachnoid hematoma, intrathecal hematoma, arachnoiditis and infection.^17^

## CONCLUSION

For patients with PDPH and a VAS score between 4 and 6 who have not responded to conservative medical treatment, an ultrasound-guided bilateral greater occipital nerve blockage is an effective treatment with fewer complications than more invasive treatment approaches. Additional controlled studies are required to establish the safe and frequent use of this method.

## Authors’ Contribution:

**FA** conceived, designed and did data collection.

**OB **editing of manuscript.

**FA, OB, UK, GK **did review and manuscript writing.

**UK** did statistical analysis 

**OB **takes the responsibility and is accountable for all aspects of the work in ensuring that questions related to the accuracy or integrity of any part of the work are appropriately investigated and resolved.
